# The beneficial role of proteolysis in skeletal muscle growth and stress adaptation

**DOI:** 10.1186/s13395-016-0086-6

**Published:** 2016-04-06

**Authors:** Ryan A. V. Bell, Mohammad Al-Khalaf, Lynn A. Megeney

**Affiliations:** Regenerative Medicine Program, Sprott Center for Stem Cell Research, Ottawa Hospital Research Institute, The Ottawa Hospital, Ottawa, ON K1H 8L6 Canada; Department of Cellular and Molecular Medicine, University of Ottawa, Ottawa, ON Canada; Department of Medicine, Division of Cardiology, University of Ottawa, Ottawa, ON Canada

**Keywords:** Proteolysis, Proteasome, Autophagy, Caspase, Muscle growth, Muscle cell differentiation

## Abstract

Muscle atrophy derived from excessive proteolysis is a hallmark of numerous disease conditions. Accordingly, the negative consequences of skeletal muscle protein breakdown often overshadow the critical nature of proteolytic systems in maintaining normal cellular function. Here, we discuss the major cellular proteolysis machinery—the ubiquitin/proteosome system, the autophagy/lysosomal system, and caspase-mediated protein cleavage—and the critical role of these protein machines in establishing and preserving muscle health. We examine how ordered degradation modifies (1) the spatiotemporal expression of myogenic regulatory factors during myoblast differentiation, (2) membrane fusion during myotube formation, (3) sarcomere remodeling and muscle growth following physical stress, and (4) energy homeostasis during nutrient deprivation. Finally, we review the origin and etiology of a number of myopathies and how these devastating conditions arise from inborn errors in proteolysis.

## Background

Maintaining protein homeostasis (proteostasis) is a critical factor in preventing cellular dysfunction and the propagation of many disease states. Proteostasis requires both protein synthesis and degradation, although the latter is more often associated with pathological states than with normal cellular functioning. The negative connotation of protein degradation is particularly evident for skeletal muscle, where excessive protein destruction is the principle mechanism behind muscle atrophy and weakness. This view is propagated by the fact that activation of proteolysis is a feature of a number of pathologies, including cancer, sepsis, uremia, acquired immune deficiency syndrome, and diabetes mellitus (reviewed in [72]). While the concern regarding the aberrant activation of proteolysis is justified in these disease states, a strictly negative association ignores the fact that the long-term viability and maintenance of any organ system will require regular protein turnover.

The necessity for properly functioning proteolytic systems in skeletal muscle is especially acute when one considers the unique requirements for this tissue. Skeletal muscles are the force-generating structures of the body and are constantly challenged by mechanical, heat, and oxidative stress. These events increase protein damage and require efficient protein turnover to maintain optimal functioning. Furthermore, skeletal muscle repair and regeneration is dependent upon the differentiation of satellite cell-derived myoblasts [13], which requires extensive remodeling and the spatiotemporal expression of myogenic factors. This, of course, requires the temporal destruction and synthesis of the appropriate proteins. These examples illustrate the necessity of proteolysis in skeletal muscle, and this review will focus on the critical nature of proteolytic systems—namely proteasome-, autophagy-, and caspase-mediated proteolysis—to sustain skeletal muscle health and development.

## Proteasome-mediated proteolysis

Perhaps the most well-known cellular proteolytic system is the ubiquitin/proteasome pathway (UPP), which is responsible for degrading the majority of cellular proteins [93]. This is a system whereby proteins meant for destruction are enzymatically tagged with the polypeptide ubiquitin via E3 ubiquitin ligases. These tagged proteins are recognized by the 26S proteasome, which is a large barrel protein complex that consists of a 20S core particle associated with two 19S regulatory subunits. The latter subunits recognize and bind the ubiquitinated proteins and begin their adenosine triphosphate (ATP)-dependent destruction within the catalytic core [119]. In the skeletal muscle, protein degradation via the proteasome is often associated with muscle atrophy. Indeed, muscle wasting is characterized by increased proteolysis via the UPP, increased ubiquitin conjugation to muscle proteins, and an up-regulation of ubiquitin-protein ligases (reviewed by [58]). However, this clear role in muscle atrophy minimizes the necessity of the UPP in maintaining normal muscle functioning. The following sections highlight the essential roles of the UPP in the skeletal muscle.

### Proteasome and muscle cell differentiation

**Fig. 1:**
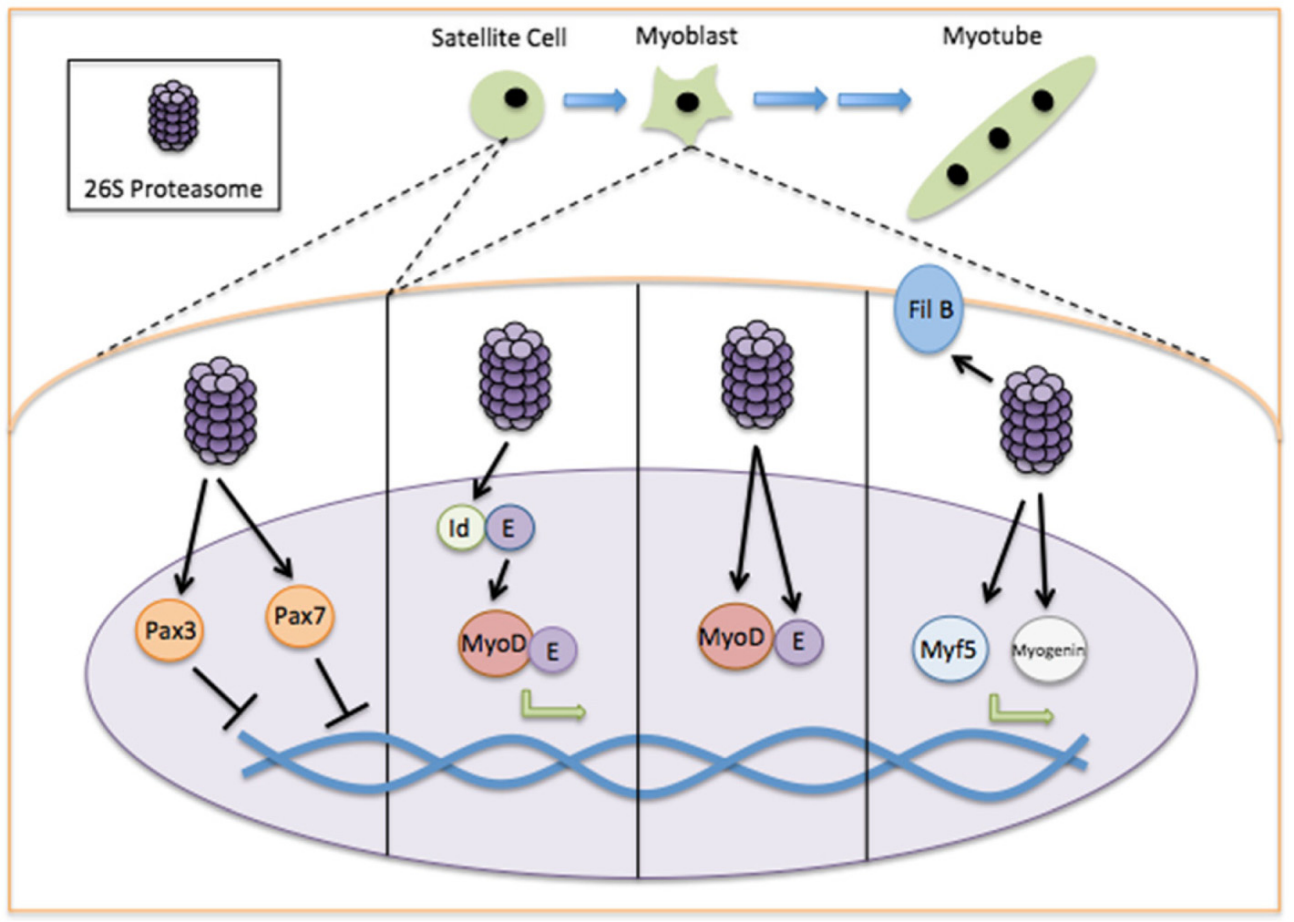
The role of the UPP in skeletal muscle cell differentiation. The fidelity of muscle cell differentiation is dependent upon the spatiotemporal expression of particular myogenic proteins. Indeed, UPP involvement in satellite cell differentiation begins with its role in the removal of Pax3 and Pax7, which maintain satellite cells in their stem cell niche. Further, the 26S proteasome appears critical for the early activation of a key myogenic factor, MyoD, through the removal of an endogenous MyoD inhibitor, Id. The continuation of the myogenic program relies on UPP-dependent degradation of MyoD and its binding partner E2A (E), as well as Myf5, myogenin, and filamin B (Fil B) during later stages of differentiation

Myogenesis is a complex process that is dependent on the spatiotemporal expression of a variety of myogenic regulatory factors. As myoblast differentiation proceeds, there is a need for timely synthesis and degradation of the appropriate myogenic proteins, which suggests a significant role for adaptive proteolysis in the myogenic process. Indeed, early studies indicated that proteasome inhibition decreased myoblast fusion and differentiation [36, 75] and prevented the degradation of key myogenic proteins, such as MyoD. MyoD degradation via the UPP was further confirmed by a series of studies [1, 48, 104, 105] and was accompanied by the revelation that many of the other proteins critical to the progression of the myogenic program were degraded in a similar manner (Fig. 1). This extends to the myogenic regulatory factors Myf5 [61], myogenin [47], Id1 (negative regulator of MyoD; [105]), E2A proteins [106], and filamin B [8]. Interestingly, both Pax3 and Pax7 are also subject to ubiquitin-mediated degradation, implying that the acquisition of differentiation competence requires proteolytic cleavage [10, 11]. In addition, recent evidence has suggested that the immunoproteasome—so called due to the interferon-γ-induced expression of three alternative proteasome β subunits—also plays a role in myogenesis, with its suppression leading to decreased myoblast differentiation [16].

The necessity of proper proteasome functioning during myoblast differentiation may extend beyond the effects on the spatiotemporal expression of transcription factors. For instance, myogenesis is a period of intense restructuring that relies on increased mitochondrial energy production. As a by-product, differentiating myoblasts produce increased levels of reactive oxygen species (ROS) and an elevated level of oxidized proteins, which must be degraded [67]. Without proper proteasome functioning, the accumulated oxidized proteins may halt the differentiation process [100] and possibly initiate apoptosis [27]. Taken together, myogenesis appears to integrate proteasome-mediated proteolysis to achieve effective myoblast differentiation.

### Proteasome and muscle growth

Recently, researchers have demonstrated that the muscle-specific knockout of an essential 26S proteasome protein, Rpt3 (also known as Psmc4), leads to a significant deficit in muscle growth and force generation in mice [52]. This sentiment is echoed in earlier publications on proteasome function in *Drosophila* muscle, where the conditional expression of a mutant proteasome β subunit (within the 20S core particle) led to the deterioration of muscle architecture [40].

**Fig. 2:**
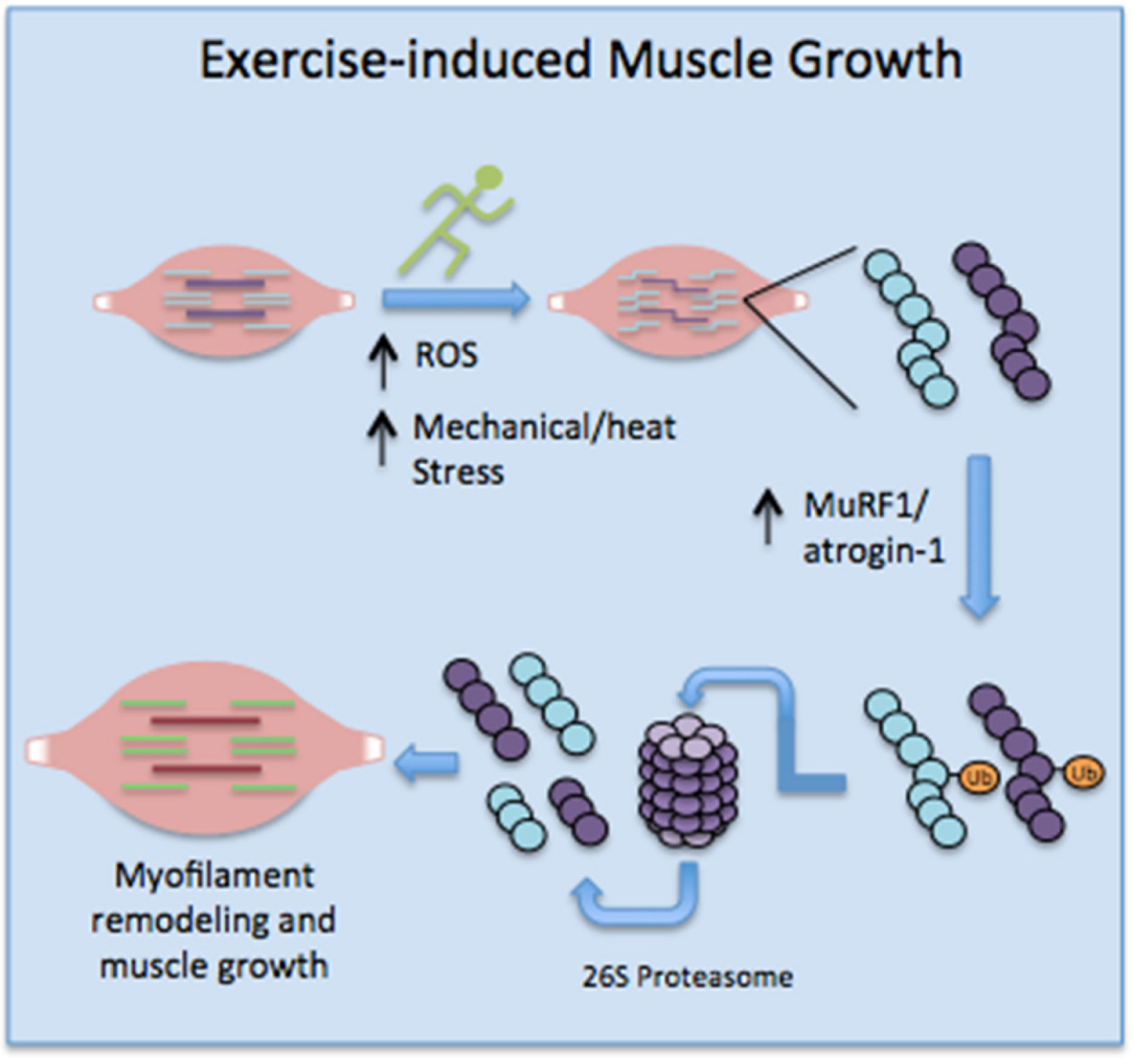
The role of the UPP in skeletal muscle growth. Exercise-induced protein damage via increased ROS/mechanical and heat stress necessitates an increase in proteasome-mediated proteolysis to rid the cells of non-functional myofibrillar proteins. This is typically dependent on a prerequisite increase in key muscle-specific ubiquitin ligases, MuRF1 and atrogin-1 (MAFbx), which ubiquitinate and target damaged proteins for degradation by the 26S proteasome. Efficient removal of damaged proteins is critical to skeletal muscle growth and remodeling following exercise

The apparent role of the UPP in muscle growth and integrity suggests that proteasome-mediated protein degradation may be important during exercise. Indeed, acute bouts of resistance exercise have been shown to increase both protein synthesis and breakdown in skeletal muscle [92]. Moreover, numerous studies have indicated that the expression of two muscle-specific ubiquitin ligase genes, muscle really interesting novel gene (RING) finger-1 (*MuRF1*) and muscle atrophy F-box (*MAFbx*; also called *atrogin-1*), increased following acute resistance exercise (reviewed by [78]), suggesting increased proteasome-mediated proteolysis post-exercise. Similarly, both acute and chronic bouts of endurance exercise appear to increase proteasome-mediated proteolysis. In the former case, a single bout of endurance running led to increased expression of MuRF1 and atrogin-1 immediately after the run (0–4 h post-exercise), suggesting increased UPP flux [64, 85]. Interestingly, chronic endurance exercise (i.e., 8-week running regimen) in mice also elicited a sustained increase in *MuRF1* expression and proteasome activity [18]. The reason for the sustained activation of the UPP as compared to untrained animals can only be speculated; however, it may stem from the increased oxidative capacity (and therefore ROS-derived protein damage) that is a characteristic of trained skeletal muscle. More recently, Baehr et al. [7] found that chronic loading of mice skeletal muscle using the functional overload model led to skeletal muscle hypertrophy that was characterized by increased protein synthesis and degradation via the UPP. However, in contrast to the study by Cunha et al. [18], this increased proteasome activity was independent of MuRF1 (and MAFbx) expression. Interestingly, recent studies have indicated that several other ubiquitin ligases may have important roles in determining skeletal muscle-associated phenotypes, including TRIM32 [54, 80], MUSA1 [98], MG53 [121], and Nedd4-1 [79]. In any case, the surge in protein breakdown following resistance and endurance exercise has been hypothesized to be adaptive, as it rids muscles of damaged proteins and facilitates myofilament restructuring and muscle growth (Fig. 2). Collectively, these studies offer an alternative function for the proteasome, for what otherwise has been largely considered to be a conveyor of muscle wasting and pathology.

## Autophagy/lysosome-mediated proteolysis

Autophagy is one of the major protein degradative pathways within virtually all cells of the body. It involves the sequestration of dysfunctional proteins or organelles in membrane bound vesicles (termed autophagosomes) and the subsequent fusion of these vesicles with lysosomes, where the encapsulated cytoplasmic material is degraded and essential biomolecules recycled [59]. Autophagy was originally identified as a form of programmed cell death and is often thought of as one of the principle mechanisms that spur muscle wasting [96]. Nevertheless, autophagy is important in maintaining healthy muscle and is critical in muscle adaptation to sublethal cellular stress. The following sections will explore the various roles of autophagy in maintaining skeletal muscle functioning, as well as the role of this process in skeletal muscle relevant stress responses.

### Autophagy and muscle mass maintenance

Several studies over the past decade have indicated that excessive autophagy aggravates muscle wasting and contributes to muscle weakness [25, 68, 111, 118, 122]. Indeed, autophagosome accumulation has been observed in nearly all myopathies [66]. However, recent evidence has indicated that basal autophagy is necessary to maintain muscle mass and prevent atrophy. Much of this evidence is derived from studies of autophagy-deficient mice, where critical autophagy-related genes have been knocked out (i.e., *Atg5* and *Atg7*). In the latter case, muscle-specific knockout of *Atg7* causes muscle cells to adopt myopathic characteristics such as misalignment of the Z-line, abnormal enlargement of mitochondria, distended sarcoplasmic reticulum (SR), and the formation of aberrant membranous structures [70]. Moreover, *Atg7*^*−/−*^ mice showed a 20–40 % age-dependent reduction in muscle fiber cross-sectional area with a corresponding decrease in force generation. A similar decrease in muscle cross-sectional area was observed in *Atg5*^−/−^ mice; however, this decrease did not appear to translate into decreased muscle performance [90]. Moreover, *Atg5*^*−/−*^ mice displayed further similarities to the *Atg7* knockout mice including the accumulation of membranous structures and the formation of protein aggregates. Taken together, these initial studies highlight the necessity for basal autophagy in muscle mass maintenance.

One of the key factors in muscle mass maintenance is the regenerative capacity of the muscle satellite cells. Recent evidence has now placed autophagy at the heart of muscle regeneration, with this process being responsible for preventing quiescent muscle stem cells from taking on a senescent state. Stem cell senescence appears to be the main culprit limiting muscle regeneration in aging mammalian muscle, which thereby suggests that efficient autophagic signaling is necessary in preventing sarcopenia [35]. Moreover, muscle stem cell activation also appears reliant on autophagy, as it is thought to provide the necessary nutrients to meet the bioenergetics demands of satellite cells transitioning from quiescence to activation [109].

In addition to the basal requirement of autophagic flux to maintain muscle mass and muscle regeneration capacity, there is a growing evidence for an essential role for autophagy in exercise-induced muscle growth. Most recently, *Redd1*^*−/−*^ (regulated in development and DNA damage responses 1; an inhibitor of mTORC1) mice display decreased autophagic flux and a significant decline in exercise capacity [87]. Indeed, the mechanical stress and the simultaneous production of ROS during physical exercise [75] increase the necessity for autophagic removal of damaged cellular components. Moreover, autophagy may be necessary during exercise-induced energy stress to provide muscle cells with an alternative energy source.

Some of the more convincing evidence for the importance of autophagy during physical exercise stem from studies of ultra-endurance runners. For instance, a recent study indicated that these runners display a 50 % decrease in FoxO3 phosphorylation (consistent with its activation and translocation into the nucleus), a fivefold increase in the phosphatidylethanolamine-conjugated microtubule-associated protein 1A/1B-light chain 3 (LC3-II) expression (indicative of increased autophagy), and a significant increase in Atg5-Atg12 complex formation (important for autophagosome formation) [45]. A study by the same group also indicated that a bout of ultra-endurance running increased skeletal muscle expression of key autophagy genes such as *Atg4b*, *Atg12*, *Gabarap1*, *LC3*, *Bnip3*, and *Bnip3l* [46]. It is likely that these studies on endurance runners represent an extreme example of skeletal muscle stress, which requires autophagy to meet energetic demands and allow for the removal of dysfunctional proteins/organelles that will inevitably accumulate with extreme contractile demands.

**Fig. 3:**
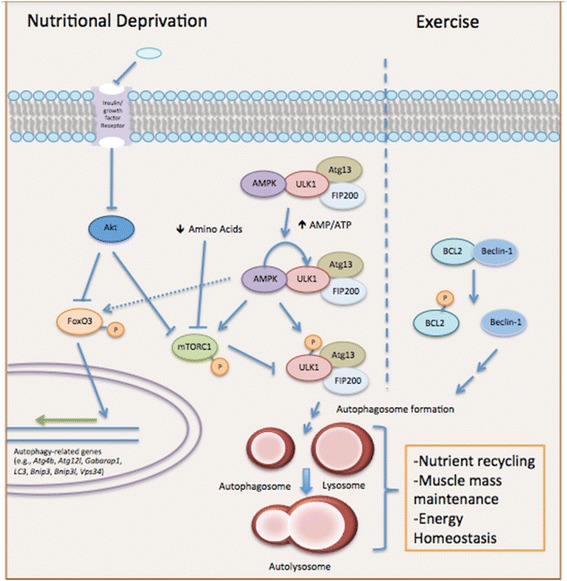
Exercise- and starvation-induced autophagy pathways and their beneficial role in muscle stress adaptation. Nutrient deprivation decreases signaling through insulin/growth factor receptors, which decreases Akt activation and allows for the AMPK-dependent phosphorylation of FoxO3. FoxO3 is then able to translocate into the nucleus and initiate the transcription of autophagy-related genes. Activated AMPK also phosphorylates mTORC1 (preventing its action on ULK1, a key autophagy-related kinase) and ULK1 to allow for efficient autophagosome formation and clearance of encapsulated material. Moreover, the lack of intake of essential amino acids further prevents mTORC1 activation and promotes autophagy induction. Taken together, these processes recycle nutrients for muscle cells and the body as a whole during lean periods. While starvation-induced autophagy is undoubtedly a part of muscle biochemistry during exercise, physical activity also activates beclin-1 through its phosphorylation-dependent release from the BCL2-beclin-1 complex. Beclin-1 is critical to autophagosome formation and the efficient clearance of damaged organelles and proteins that arise from physical stress

In juxtaposition to the studies on ultra-endurance running, the effects of acute and chronic exercise on the autophagic response in skeletal muscle cells appear to be inconclusive. With respect to acute bouts of exercise, one investigation demonstrated that murine skeletal muscle autophagosome formation increased after ~30 min of treadmill running. Furthermore, the transgenic mice used in this study, which lack the ability to activate exercise-induced autophagy through the release of beclin-1 from the B cell lymphoma 2 (BCL2)-beclin-1 complex (Fig. 3), display a marked reduction in endurance during acute treadmill running [41]. Thus, this study indicates not only that autophagy is induced during short-term exercise but that the lack of autophagic flux hinders muscle performance. Grumati and colleagues [39] also found that a 60-min bout of treadmill running was capable of increasing autophagic flux in murine skeletal muscles, as evidenced by increased LC3 lipidation (i.e., conversion of LC3-I to LC3-II via phosphatidylethanolamine conjugation). Most recently, an acute bout of exercise (60–90 min in duration) caused a significant increase in autophagic signaling in both mouse and human skeletal muscle [74, 109]. One potential aspect of increased autophagy following acute exercise appears to be in mediating mitochondrial turnover (termed mitophagy), which was recently shown to be coordinated, in part, by the transcriptional coactivator peroxisome proliferator-activated receptor-γ coactivator-1α (PGC-1α) [116]. In contrast to these studies, Kim et al. [50] demonstrated that 50 min of treadmill running decreased the expression of autophagy-related genes (i.e., *LC3*, *beclin-1*, and *Atg7*), a few hours post-exercise. It is important to note that the exercise regimens between these studies were not identical and varied in aspects such as speed, treadmill inclination, and time. These are likely significant factors given the recent research by Schwalm et al. [99] that indicates that exercise intensity may be the crucial factor in determining the induction of autophagy. Moreover, the differences in the level of autophagic induction may stem from intrinsic differences in muscle glycogen reserves and pre-exercise energy status (i.e., AMP to ATP ratio and the corresponding activation state of AMP-dependent protein kinase (AMPK)). Indeed, it is reasonable to assume that a threshold may exist during aerobic exercise where muscle cells engage in autophagy-mediated breakdown of damaged organelles/proteins to ensure normal functioning and energy homeostasis.

Similar to the above situation, skeletal muscle autophagic flux following chronic exercise appears to be quite variable. For instance, analysis of the tibialis anterior (TA) muscle in mice that were allowed to spontaneously exercise on a running wheel for 3 months did not show any evidence of LC3 lipidation [39]. Conversely, investigation of the plantaris muscle in mice subjected to 4 weeks of voluntary running displayed increased LC3 lipidation, decreased p62 protein content (indicating greater autophagic flux), and increased expression of a number of autophagy-related proteins (i.e., *Atg6*, *LC3*, and *Bnip3*) [62]. A recent study confirmed these findings with basal autophagy in mice plantaris muscle being induced after a 20-week period of voluntary exercise [108]. One major difference between these two studies and the previous study on the TA muscle is the difference in muscle types analyzed. Plantaris is composed of mixed muscle fiber types (glycolytic/oxidative) while the TA is a mainly composed of glycolytic (type II) fibers [3, 110]. Muscles with mixed muscle fiber types often become more oxidative with chronic aerobic exercise [108], and thus, an induction of autophagy may be necessary as an adaptive mechanism to deal with increased oxidative stress and mitochondrial turnover. Recently, a study of the rectus femoris muscle (consisting of mixed fiber types) following 2 months of exercise training displayed increased LC3 expression, decreased p62 expression, and an overall increase in autophagic flux [44]. Similarly, analysis of rat soleus muscle (composed of mainly oxidative muscle fibers) after chronic exercise (1 h/day, 6 days/week, for 8 weeks) showed increases in a variety of autophagy-related genes such as *Atg7*, *beclin-1*, and *LC3* [28]. These rats also displayed mitochondrial dysfunction and the induction of an oxidative stress response, which may explain the increases in autophagy markers.

### Autophagy and nutrient stress response

Nutrient deprivation is one of the most potent activators of autophagy, and while this generally promotes muscle wasting, the process appears necessary in times of energy stress to supply the body with catabolic substrates to allow continued functioning [91]. Indeed, in vivo analysis has indicated that there is a significant increase in autophagosome formation in skeletal muscle following a 24-h period of starvation in mice [73]. Interestingly, autophagosome formation differed between muscle fiber types, with fast-twitch muscle fibers showing a significantly greater autophagic response as compared to slow-twitch muscle fibers [73]. Previous studies on the overall protein degradation have indicated that slow-twitch muscle fibers are more resilient than fast-twitch fibers during starvation [31, 60], which may be adaptive given the importance of slow-twitch fibers in maintaining posture.

In recent years, significant strides have been made in determining the mechanism by which nutrient deprivation triggers skeletal muscle autophagy (Fig. 3). Early evidence indicated that starvation-induced autophagy was mediated via the Akt/FoxO3 axis [68, 122]. Specifically, the lack of growth factor- and insulin-dependent signaling during nutrient deprivation suppresses Akt activation and subsequent FoxO3 phosphorylation (at Akt specific sites). This then allows for the nuclear translocation of FoxO3 and the transcriptional initiation of autophagy-related genes, such as those essential for autophagosome formation (*Atg12l*, *Atg4b*, *Gabarapl1*, and *LC3*) and the regulation of autophagy (*Bnip3*, *Bnip3L*, and *Vps34*) [68, 122]. Further research indicated that transcriptional activation of FoxO3 was promoted by AMP-dependent protein kinase (AMPK)-dependent phosphorylation. AMPK also appears to bind and regulate Unc-51 like autophagy activating kinase 1 (ULK1), which is important in the induction of autophagy and the formation of autophagosomes. Sanchez et al. [95] found that AMPK and ULK1 are interacting partners during periods of nutrient abundance, with nutrient stress causing their dissociation and an increase in ULK1 activity (through AMPK-dependent phosphorylation), thereby aiding in autophagosome biogenesis.

Surprisingly, the aforementioned studies generally found that the nutrient-responsive kinase, mammalian target of rapamycin (mTOR), contributed little to the induction of autophagy in skeletal muscle during starvation. Zhao et al. [122] found that inhibition of mTOR complex 1 (mTORC1) in C2C12 myotubes by rapamycin only resulted in a slight increase in autophagy, while inhibition of the upstream kinase, Akt, via API-2, induced an autophagic response that was fivefold greater than that with rapamycin treatment. Similarly, Mammucari and colleagues [68] found that rapamycin inhibition and knockdown of mTOR had little effect on the induction of lysosomal proteolysis. This same study also indicated that the RNAi-mediated knockdown of the rapamycin-insensitive mTOR complex (mTORC2) did induce FoxO3 translocation into the nucleus. In contrast to these findings, a recent study indicated that constitutive activation of mTORC1 in the skeletal muscle of tuberous sclerosis 1 (TSC1)-deficient mice inhibited autophagy despite AMPK and FoxO3 activation [12]. In these mice, active skeletal muscle mTORC1 was shown to phosphorylate ULK1 and thereby prevent the AMPK-dependent phosphorylation of ULK1 that is necessary for autophagosome formation [49]. Indeed, rapamycin administration or institution of a ULK1 mutant that was insusceptible to mTORC1 phosphorylation was sufficient to restore autophagic flux. While this study does not discount the importance of AMPK/FoxO3 signaling, it does indicate that inhibition of mTORC1 may be necessary for efficient autophagy induction. Interestingly, these two signaling pathways have been linked, with AMPK being capable of phosphorylating mTORC1 and disrupting any interaction with ULK1 [95]. Thus, the discrepancies in the aforementioned studies may suggest that an initial decrease in ATP to AMP ratio during nutrient deprivation may activate AMPK, which will subsequently initiate FoxO3 signaling and, concurrently, inhibit mTORC1 to allow ULK1 function and autophagy to be initiated.

An additional aspect of nutrient deprivation that directly affects mTOR and thereby autophagic signaling is the lack of intake of essential amino acids (e.g., leucine). This deficiency prevents amino acid-induced mTORC1 translocation to lysosomal membranes where mTORC1 would be activated through an interaction with Rag GTPases and the ragulator protein complex [94]. Given that growth factor and amino acid-dependent signaling converge at the same point (i.e., mTOR), it is likely that these two signaling pathways act in concert during nutrient deprivation to activate autophagy and recycle essential building blocks.

### Autophagy and metabolism

The link between autophagy and metabolism has been well established based on its function in recycling damaged proteins and organelles during energy stress. However, recently, autophagy has been suggested to play a role in carbohydrate and lipid metabolism. In the latter case, evidence has emerged that autophagic activity is inversely correlated with intramyocellular triglyceride levels in morbidly obese patients following bariatric surgery [55]. These researchers also found that L6 rat myocytes cultured with free fatty acids showed increased lipid accumulation and cell death when autophagy was inhibited and decreased lipid accumulation when autophagy was activated. These results clearly imply a role for autophagy in lipid metabolism; however, further experiments will be needed to elucidate its exact role.

Skeletal muscle carbohydrate metabolism also appears to be affected by autophagic flux, with the strongest evidence emerging from the study of various muscle pathologies. For instance, in patients with Danon disease, glycogen granules accumulate in autophagosomes that are unable to fuse with lysosomes for proper clearance, which leads to muscle weakness [82]. Interestingly, patients with Pompe disease exhibit a defect in glycogen breakdown within lysosomes, and muscle-specific inhibition of autophagic transport of glycogen to lysosomes confers some therapeutic benefits [33, 90]. Autophagy may also be linked to glucose homeostasis. Mice deficient in exercise-induced autophagy (i.e., inhibited release of beclin-1 from the BCL2-beclin-1 complex) also show decreased insulin sensitivity and impaired redistribution of glucose transporter 4 (GLUT4) in skeletal muscle cells in response to acute exercise [41]. Moreover, this study demonstrated that impaired exercise-induced autophagy negated the exercise-mediated benefits to glucose tolerance in obese mice. In direct opposition to this study, muscle-specific knockout of *Atg7* (suppressing autophagy) led to increased lipid oxidation and decreased high-fat diet-induced insulin resistance [51]. While in not identical studies, the discrepancies do indicate the need for further investigation regarding the role (if any) of autophagy in insulin sensitivity and glucose homeostasis. These studies aside, it appears likely that autophagy plays an important role in lipid and glycogen metabolism within skeletal muscle cells; however, the underlying mechanisms remain unclear.

### Autophagy dysfunction and disease

The necessity for proper autophagic flux in maintaining skeletal muscle functioning is most evident when one considers the pathologies that accompany a dysfunctional autophagic process. The aforementioned Danon disease is a prime example of myopathy caused by disturbed autophagic flux, with impaired autophagosome/lysosome fusion leading to muscle weakness and various extra-muscular effects. Similarly, there are a variety of other related diseases that likely develop, at least in part, due to defects in autophagosome/lysosome fusion. These include Vici syndrome [17], X-linked myopathy with excessive autophagy, adult-onset vacuolar myopathy with multiorgan involvement, and infantile vacuolar autophagic myopathy [81]. Moreover, muscle disorders can arise from the defects in the autophagic clearance of disease-causing molecules. For instance, in sporadic inclusion body myositis, one of the principle mechanisms for its initiation is the accumulation of amyloid precursor protein and its fragment, β-amyloid in muscle cells [5]. These two proteins associate with LC3 in cultured muscle cells and biopsied degenerating muscle fibers, which suggests that they may be cleared through autophagy [65]. Similarly, limb girdle muscular dystrophy type 2B and Miyoshi myopathy arise due to the aggregation of mutant dysferlin (typically a sarcolemmal protein) in the endoplasmic reticulum. Activation of the stress-induced autophagic pathway increases mutant dysferlin degradation, while a blockade in autophagy (i.e., through Atg5 depletion) promotes aggregate formation [32]. Accumulation of mutant filamin C, an actin-binding protein that functions at the Z-disk, also triggers protein aggregation and the development of myofibrillar myopathy. This accumulation is thought to be due in part to a disruption in chaperone-assisted selective autophagy (CASA) [53]. Additionally, skeletal muscles of one of the more detrimental myopathies, Duchenne muscular dystrophy, were recently found to display impaired autophagy. This was evidenced by decreased LC3 lipidation, the accumulation of damaged organelles, and decreased expression of autophagy-related genes [20]. Lastly, disrupted autophagy is thought to play an important role in the progression of myopathies derived from mutated collagen VI genes, such as Ullrich congenital muscular dystrophy and Bethlem disease (reviewed in [97]). These genes encode for a key skeletal muscle extracellular matrix (ECM) protein [56], and studies of skeletal muscle *Col6a1*^*−/−*^ mice and humans have indicated that this defect leads to the formation of abnormal mitochondria and SR, as well as the initiation of apoptosis [4, 43]. Subsequent experiments revealed that these aberrant organelles were the result of a malfunction in the autophagic process, specifically impairment in autophagosome formation [38]. Indeed, nutritional or pharmacological reactivation of autophagy attenuated the dystrophic phenotype in *Col6a1*^*-/-*^ mice. Taken together, these myopathies further illustrate the importance of skeletal muscle autophagy in maintaining normal muscle function and identifying the constellation of relevant autophagy/vacuolar proteases that manage this process will be a high priority.

## Caspase-mediated proteolysis

Caspases (cysteine-aspartic proteases) are a family of proteolytic enzymes that are most commonly known for their role in initiating apoptosis. Caspases are typically classified as initiator caspases (caspase-2, caspase-8, caspase-9, caspase-10) or effector caspases (caspase-3, caspase-6, caspase-7), with the former being responsible for activating the effector caspases. The role of caspases in apoptosis and the association of these proteases with various forms of muscle atrophy have reinforced the negative stereotype of this protein family, as conduits of cell destruction [26]. Despite the prevailing death-centric view, caspase-mediated signaling events have been linked to a diverse array of vital cell tasks, which are independent of inducing apoptosis [24].

### Caspases and satellite cell commitment

Satellite cell commitment to the muscle cell lineage is an essential step in muscle growth and regeneration. Caspase activity appears to be intimately involved in this process as recent studies have indicated that caspase 3 activity directly limits satellite cell self renewal, by cleaving and inactivating the paired box transcription factor Pax7 [23, 83]. Pax7 is essential to maintain the satellite cell niche and must be removed for satellite cells to acquire differentiation competence [84]. Given that Pax7 is subject to both caspase and ubiquitin targeted degradation, a reasonable conjecture may be that these processes work in tandem to ensure the acquisition of a differentiation competent state. One could envision the initial caspase-dependent cleavage of Pax7, followed by ubiquitination and removal of Pax7 fragments. How and whether these proteolytic systems engage in crosstalk will require further experimentation.

### Caspases and skeletal myoblast differentiation

**Fig. 4:**
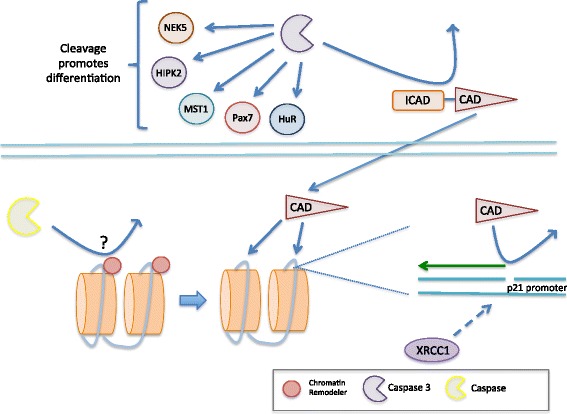
The role of caspases in skeletal myoblast differentiation. Caspase 3 has a multifaceted role in regulating myogenesis. It is responsible for the proteolytic cleavage of the transcription factor Pax7, which maintains satellite cells in their stem cell niche and prevents myoblast differentiation. Moreover, caspase 3 cleaves the promyogenic kinases MST1, HIPK2, and NEK5 to promote myogenesis. The posttranscriptional regulator, HuR, is also cleaved by caspase 3 and is necessary for muscle fiber formation. Additionally, preliminary evidence (unpublished) suggests that the myogenic differentiation program appears to rely on the caspase-mediated cleavage of chromatin remodeling proteins to increase DNA accessibility for CAD (activated by caspase 3 cleavage of ICAD), which produces DNA strand breaks that are critical to regulating myogenic gene expression. For instance, CAD cleavage of the p21 promoter stimulates p21 expression, which is essential for cell cycle arrest and terminal differentiation. The CAD-derived DNA strand breaks require rapid resolution, which is mediated by the base excision repair protein XRCC1. This mending of DNA strand breaks is necessary to stabilize the genome and ensure the fidelity of the myogenic differentiation program

Skeletal myoblast differentiation and the early steps in apoptosis possess a remarkable number of similarities. For instance, actin fiber disassembly/reorganization and phospholipid reorientation are features of both apoptosis [15] and differentiation [34, 88, 117]. Moreover, these seemingly conflicting cell fates both require increased activity of select matrix metalloproteinases [69, 120]. These similarities spurred a controversial hypothesis that suggested muscle cell differentiation and apoptosis may utilize overlapping signaling cascades, a supposition that was initially addressed in 2002. Here, Fernando et al. [29] demonstrated that transient caspase-3 activity is required for myoblast differentiation and that this non-death activity is mediated in part through the cleavage activation of the Ste-20 like kinase, macrophage stimulating 1 (MST1). Subsequent studies have established that the key elements of the intrinsic mediated cell death pathway are fully conserved to engage caspase 3 during myoblast differentiation [37, 77]. Once activated, caspase 3 targets multiple substrates to engage the differentiation program (Fig. 4). These substrates include promyogenic kinases such as MST1, HIPK2, NEK5 [19, 29, 101], the posttranscriptional regulatory protein ELAV-like protein (HuR) [6], and caspase-activated DNase (CAD), where the latter is activated by cleaving and removing its nascent inhibitor inhibitor of caspase-activated DNase (ICAD) [57]. CAD promotes myoblast differentiation by inflicting transient DNA strand breaks at the promoters of critical regulatory factors such as the cell cycle inhibitor p21, an event that leads to *p21* induction [2, 57]. Interestingly, CAD-sensitive strand breaks are detectable throughout differentiating myonuclei, suggesting that the DNase may engage a genome wide reprogramming event to alter the expression of a large number of gene targets. It is important to note that these DNA strand breaks need to be resolved quickly for proper muscle cell differentiation, and recent evidence has indicated that the base excision repair protein, XRCC1, is a key player in mending CAD-dependent breakage events [2].

In addition to augmenting myogenic gene expression, caspase 3 regulates other key features of myogenesis. For instance, phosphatidylserine receptor-mediated caspase 3 activation has been reported to enhance myoblast fusion to existing myofibers. The relevant caspase substrates have not been characterized in this process, yet the protease activity appears to be a critical step in effecting muscle maturation and regeneration via cell fusion [42].

These observations confirm that caspase 3 acts at multiple points to secure the differentiation program, yet the mechanism that spurs caspase activity at these specific temporal junctures remains unknown. Satellite cell activation has been reported to trigger engagement of the extrinsic mediated cell death pathway such as the Fas-associated protein with death domain (FADD) receptor [14], yet the requisite initiator caspase for this pathway, caspase 8, does not appear to be appreciably activated at this stage [37]. Given the central role of caspase 3 signaling in the differentiation process, there is a significant need to identify (1) the pathways that engage the activation of this protease, (2) the full range of substrates that facilitate its capacity to induce differentiation, and (3) the mechanisms that restrain protease activity and direct it to a non-death cell function.

### Caspases and skeletal muscle adaptation

The central role of caspase 3 in directing myoblast differentiation suggests that this protease and its cognate signaling pathways may retain non-death functions within fully formed myofibers. Notably, Wang et al. [113] have reported that caspase 3 targets and cleaves proteins that lead to acetylcholine receptor dispersal in postsynaptic membranes, a key step in the development of the neuromuscular junction. Skeletal muscle fibers express the repertoire of caspase regulatory proteins, and caspase 3 has been linked to a wide array of remodeling activities in various cell lineages, including postsynaptic remodeling in neurons and cardiomyocyte hypertrophy [24, 86]. The link between caspase 3 and cardiomyocyte hypertrophy is of particular interest as this form of cell growth is an adaptive stress response. As cardiac and skeletal muscle cells share a remarkable overlap in regulatory gene expression, sarcomere assembly/content etc., it is not unreasonable to suggest that caspase 3 activity may also manage the hypertrophy of skeletal muscle fibers through discrete proteolytic targeting steps.

How caspase 3 activity translates to a beneficial stress adaptation in skeletal muscle fibers is currently unknown. One probable mechanism may involve direct communication between caspase 3 and other proteostatic control mechanisms within the muscle fiber (i.e., caspase signaling may serve to control both proteasome and autophagy-related signaling during muscle adaptation). To date, over 500 physiologic substrates have been identified for caspase 3/7, as such the ability of an effector caspase to target and integrate regulatory control over disparate proteolytic mechanisms is an entirely probable event. In *Drosophila* oogenesis, the effector caspase equivalent, death caspase-1 (DCP-1), has been shown to promote autophagy flux by cleaving and inhibiting the key autophagy suppressing protein SesB [22]. Whereas autophagy has been generally associated with muscle atrophy/wasting, a number of studies have shown that resistance trained skeletal muscle is associated with enhanced autophagic flux [62, 114]. The observations in *Drosophila* have established the existence of a caspase-directed autophagy signal, whether skeletal muscle utilizes a similar beneficial regulatory cascade will require further investigation. Simultaneous caspase activation and proteosome signaling in skeletal muscle are understood to have generally negative outcomes, associated with wasting and atrophy in a variety of disease settings [26, 71, 102]. Nevertheless, caspase 3 has been demonstrated to target and cleave subunits of the 19S proteasome (Rpt2 and Rpt6), leading to an obligatory increase in proteasome activity during myoblast differentiation [112]. Mutation of the respective caspase 3 cleavage sites in Rpt2 and Rpt6 resulted in failure to up-regulate the 19S proteasome, with a profound block in the differentiation program. Clearly, the uncontrolled engagement of this signaling interaction would have dire consequences for myofiber viability, yet one can easily envision that a transient activation of these proteases may act to remodel the ultrastructure of the myofiber in response to physiologic demands.

What remains unknown are the biochemical controls that allow for the activation of effector caspases, while avoiding induction of cell death/apoptosis. As noted above, caspase-mediated cell differentiation is a broadly conserved mechanism that spans all cell lineages and is extant from worms to humans [24]. The key distinction between death and non-death caspase function is the duration of the signaling cascade, where cell death is characterized by sustained caspase 3/7 activity, non-death responses by a transient activity pattern [23, 37, 77]. A probable loci of caspase control may reside with the inhibitor of apoptosis (IAPs), a class of RING Finger domain proteins that target and inhibit caspase activity by both structural blockade and through self directed ubiquitination of the IAP/caspase complex [21, 63, 107]. One IAP, X-linked inhibitor of apoptosis (XIAP), has been investigated for its capacity to modify caspase activity during myoblast differentiation, with studies supporting a role for XIAP in this regard [77, 103] and a study that concludes otherwise [9]. The additional IAPs such as cIAP1 and cIAP2 may provide similar levels of control yet they have not been investigated in this context. Finally, it is relevant to note, that while protein interactions with the effector caspases may direct the activation of these proteases for non-death outcomes, the ultimate arbiter of control may originate with the initiation signal per se. Activation of intrinsic and/or extrinsic cell death signal cascades during non-apoptotic cell adaptation(s) are themselves uniformly transient [30, 115], suggesting that external physiologic inputs may be the deciding factor in managing effector caspase-mediated outcomes in any tissue, including skeletal muscle.

## Conclusions

In addition to its role in simply maintaining proteostasis, proteolysis is an essential part of the production of new skeletal muscle fibers and adapting muscle fibers to cellular stress. Current research clearly indicates a critical role of protein degradation in the regulation of the myogenic differentiation program, ensuring timely protein expression for myoblast differentiation while also mediating myoblast fusion and myotube formation. The mature muscle fiber then relies on appropriate protein degradation to rid the cell of damaged proteins from the mechanical and oxidative stress that accompanies the force-bearing/force-generating function of skeletal muscle. Additionally, during nutrient deprivation the organism depends on skeletal muscle proteolysis to maintain whole-body energy homeostasis. Not surprisingly, defects within these proteolytic systems often result in the development of myopathic conditions. Thus, future work in this field should focus on delineating the mechanistic details of protease function in healthy skeletal muscle. In our opinion, this information is essential, as indiscriminate targeting of proteolytic pathways as a means to treat muscle atrophy may engender more harm than benefit.

## Abbreviations

AMP: adenosine monophosphate
AMPK: AMP-dependent protein kinase
ATP: adenosine triphosphate
BCL2: B cell lymphoma 2
CASA: chaperone-assisted selective autophagy
DCP-1: death caspase-1
ECM: extracellular matrix
FADD: Fas-associated protein with death domain
GLUT4: glucose transporter 4
HuR: ELAV-like protein
HIPK2: homeodomain-interacting protein kinase 2
IAP: inhibitor of apoptosis
LC3: phosphatidylethanolamine-conjugated microtubule-associated protein 1A/1B-light chain 3
MAFbx: muscle atrophy F-box
MST1: macrophage stimulating 1
mTOR: mammalian target of rapamycin
MuRF1: muscle really interesting novel gene (RING) finger-1
NEK5: NIMA-related kinase 5
PGC-1α: peroxisome proliferator-activated receptor-γ coactivator-1α
ROS: reactive oxygen species
SR: sarcoplasmic reticulum
TA: tibialis anterior
TSC1: tuberous sclerosis 1
ULK1: Unc-51 like autophagy activating kinase 1
XIAP: X-linked inhibitor of apoptosis
