# Erratum to: The beneficial role of proteolysis in skeletal muscle growth and stress adaptation

**DOI:** 10.1186/s13395-016-0090-x

**Published:** 2016-05-04

**Authors:** Ryan A. V. Bell, Mohammad Al-Khalaf, Lynn A. Megeney

**Affiliations:** Regenerative Medicine Program, Sprott Center for Stem Cell Research, Ottawa Hospital Research Institute, The Ottawa Hospital, Ottawa, ON K1H 8L6 Canada; Department of Cellular and Molecular Medicine, University of Ottawa, Ottawa, ON Canada; Department of Medicine, Division of Cardiology, University of Ottawa, Ottawa, ON Canada

Following the publication of the original article [1] the author notified us of an error in the manuscript, which needed to be corrected. The error appears in the following sentence, which can be found under the sub-heading “Caspases and skeletal myoblast differentiation”:

"Here, Fernando et al. [29] demonstrated that transient caspase-3 activity is required for myoblast differentiation and that this non-death activity is mediated in part through the cleavage activation of the **Ste**-**20 like kinase, macrophage stimulating 1 (MST1)"**

However, this sentence has now been corrected on the BioMed Central website, and now reads as follows:

"Here, Fernando et al. [29] demonstrated that transient caspase-3 activity is required for myoblast differentiation and that this non-death activity is mediated in part through the cleavage activation of the **mammalian sterile 20-like kinase-1, MST1"**
